# Virtual immunohistochemistry for ER and PR prediction in breast cancer histopathology: a two-stage conditional generative framework

**DOI:** 10.3389/fonc.2026.1867968

**Published:** 2026-09-16

**Authors:** Hiba Alzoubi, Abdulla Shatarah, Abdulrhman Sanajleh, Maher Sughayer

**Affiliations:** 1 Department of Basic Pathological Sciences, Faculty of Medicine, Yarmouk University, Irbid, Jordan; 2 Cirrusgo, Amman, Jordan; 3 Faculty of Medicine, Yarmouk University, Irbid, Jordan; 4 Department of Pathology, King Hussein Cancer Center, Amman, Jordan

**Keywords:** breast cancer, computational pathology, conditional generative adversarial network, digital pathology, estrogen receptor, progesterone receptor, virtual immunohistochemistry

## Abstract

**Background:**

Immunohistochemistry (IHC) remains the gold standard for assessing key breast cancer biomarkers such as estrogen receptor (ER) and progesterone receptor (PR). However, the staining process is resource-intensive, time-consuming, and dependent on specialized laboratory infrastructure. While recent advances in computational pathology have demonstrated the feasibility of predicting biomarker status directly from hematoxylin and eosin (H&E) stained tissue, most models lack visual interpretability, limiting clinical trust and adoption.

**Methods:**

We propose a two-stage deep learning pipeline for virtual immunohistochemistry. In Stage 1, a regression-based convolutional neural network predicts continuous ER/PR positivity from H&E image patches trained using a mean-squared-error objective; an Edge-Focused Loss was explored as an alternative but was not adopted. In Stage 2, the predicted biomarker percentage is incorporated as a spatial condition into a conditional generative adversarial network (cGAN), enabling the synthesis of virtual IHC images. The framework was evaluated on the IHC4BC dataset using 5-fold patient-level cross-validation.

**Results:**

The predictive model achieved a mean absolute error (MAE) of 9.14% for ER and 6.10% for PR under 5-fold patient-level cross-validation; treated as a clinical positive/negative classifier it reached an area under the ROC curve of 0.92 for ER and 0.74 for PR. The generative stage produced visually plausible virtual IHC images with biologically meaningful nuclear staining patterns. Although pixel-based metrics (SSIM and PSNR) favored the unconditioned baseline, domain-specific analysis of the deconvolved DAB channel showed substantially improved biomarker-specific staining fidelity for the conditional framework. For PR, the correlation between the generated DAB signal and ground-truth positivity increased from 0.32 (unconditioned baseline) to 0.61 in the deployed pipeline, reaching 0.82 under oracle conditioning, illustrating both the benefit of conditioning and the residual error propagated from Stage 1.

**Conclusion:**

This proof-of-concept study presents a dual-output computational framework that integrates quantitative biomarker prediction with interpretable virtual staining. The proposed approach may support preliminary biomarker assessment and workflow prioritization, particularly in resource-limited settings. However, external validation and domain-specific evaluation metrics are required before clinical application.

## 1 Introduction

Breast cancer remains one of the most frequently diagnosed malignancies globally, accounting for approximately 2.3 million new cases annually according to the World Health Organization (1). Precise molecular profiling is essential for determining effective targeted therapies and guiding clinical decision-making. In routine clinical pathology, the quantification of key biomarkers—specifically the Estrogen Receptor (ER) and Progesterone Receptor (PR)—is heavily reliant on Immunohistochemistry (IHC) (2). While IHC serves as the diagnostic gold standard by physically staining specific cellular proteins to reveal their presence and distribution within tissue, the process is inherently resource-intensive, expensive, and time-consuming (3). Furthermore, the reliance on specialized chemical reagents, calibrated laboratory equipment, and trained histotechnologists creates a significant bottleneck in diagnostic workflows, particularly in resource-constrained or developing healthcare settings where timely biomarker evaluation is critical for patient outcomes (4).

To circumvent these physical limitations, the field of computational pathology has increasingly turned to Deep Learning (DL) to predict molecular biomarker statuses directly from ubiquitous and inexpensive Hematoxylin and Eosin (H&E) stained whole-slide images (5, 6). Over the past decade, Convolutional Neural Networks (CNNs), Vision Transformers (ViTs), and weakly supervised learning paradigms have demonstrated remarkable success in extracting sub-visual morphological features from H&E slides to infer molecular properties such as hormone receptor status, HER2 expression, microsatellite instability, and even genomic subtypes (7, 8). However, conventional predictive models in this domain often suffer from a critical usability flaw: they operate as “black boxes”. These models typically output a singular classification label or regression float without providing spatial or visual justification for their prediction. In clinical practice, pathologists and oncologists are hesitant to trust a standalone AI-generated number without visual corroboration (9). To establish clinical trust, medical professionals require interpretable visual evidence demonstrating *where* and *how* the AI is identifying biomarker positivity within the tissue architecture (10).

Concurrently, the concept of “virtual staining” has gained considerable traction, whereby deep generative models are employed to translate standard H&E-stained tissue into specialized IHC stains computationally (11, 12). Representative standard H&E images and their corresponding true clinical IHC images are shown in **Figure 1**. Generative Adversarial Networks (GANs), conditional diffusion models, and various image-to-image translation frameworks have been applied to synthesize IHC representations from H&E inputs (13, 14). While these generative approaches excel at mimicking the general style and texture of IHC stains, they systematically struggle with target-specific quantitative accuracy for dense biomarkers like ER and PR. This arises because the morphological features visible in H&E alone may not contain sufficient information to deduce molecular expression rates, leading unconditioned generators to arbitrarily stain all visible tumor structures, producing visually convincing but clinically inaccurate outputs (15).

**Figure 1:**
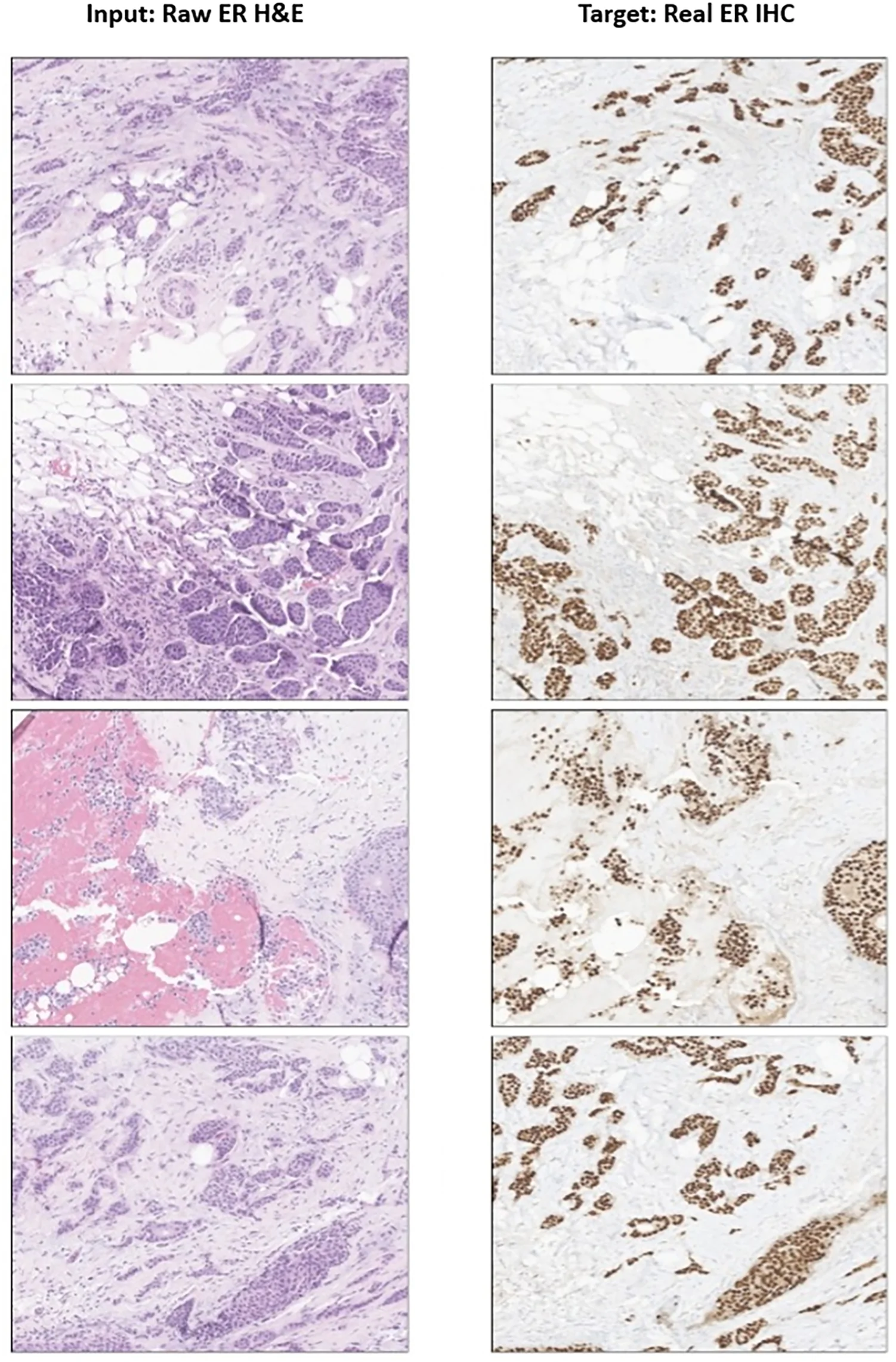
Standard H&E versus true clinical IHC.

To address both the resource constraints of physical staining and the interpretability deficit of traditional predictive AI, we propose a novel, two-stage end-to-end machine learning pipeline for “Virtual Immunohistochemistry.” Rather than relying solely on abstract numerical outputs, our architecture bridges the gap between quantitative prediction and qualitative visual verification. In Stage 1, a regression-based CNN analyzes the raw H&E image to predict the precise continuous percentage of biomarker positivity. In Stage 2, this predicted float value is embedded as a spatial condition map into a conditional generative adversarial network (cGAN), guiding the generator to synthesize a plausible IHC appearance consistent with the predicted positivity. Our core contributions in this paper are as follows:

1. Two-Stage Generative Pipeline: We introduce a dual-output framework that translates standard H&E histopathology into both a quantitative clinical percentage and a highly interpretable, synthetically generated IHC visual mapping for ER and PR biomarkers.
2. Calibrated Continuous Biomarker Regression: We frame biomarker estimation as continuous regression that addresses the spatial pooling limits of standard CNN regressors. The adopted mean-squared-error objective reduces boundary errors, substantially reducing zero-boundary hallucinations on healthy tissue and achieving a cross-validated MAE of 6.10% (PR) to 9.14% (ER) with strong clinical positive/negative classification (ER AUC 0.92). A custom Edge-Focused Loss was explored but conferred no advantage over this objective (Section 4.1).
3. Condition-Driven Virtual Staining: We demonstrate that incorporating a continuous biomarker prediction as a fourth-channel spatial condition in a conditional GAN enables quantitatively guided virtual staining, mitigating the “blind painting” limitation of conventional image-to-image translation models and producing synthetic stains that better reflect biomarker-specific spatial distributions.

Unlike prior approaches that perform either biomarker prediction or H&E-to-IHC translation in isolation, our framework links the two: the continuous positivity estimated in Stage 1 is propagated as an explicit quantitative condition that governs Stage 2 synthesis. This converts virtual staining from an unconstrained appearance-matching task into a quantitatively guided rendering task and yields a single pipeline that is simultaneously interpretable through visual staining maps and quantitative through calibrated biomarker estimates.

## 2 Related work and literature review

The intersection of deep learning and computational pathology has seen significant advancements, particularly in the prediction of molecular biomarkers and the synthetic generation of histochemical stains. Our two-stage architecture builds upon and addresses the limitations of several primary domains of research. Recent reviews and studies in breast cancer computational pathology highlight both the rapid progress of deep learning approaches and the persistent challenges of dataset diversity, model generalization, and clinical integration (16–19). While these studies encompass virtual staining, biomarker prediction, and H&E-based classification tasks, they collectively emphasize the need for robust and clinically interpretable AI frameworks. To contextualize our contribution within this evolving landscape, a structured comparison of representative recent methods, including datasets, approaches, reported results, and limitations, is provided in **Table 1**.

**Table 1:** Comparison of representative recent methods for biomarker prediction and virtual immunohistochemistry.

Study	Task	Dataset	Key method	Reported result	Main limitation
Akbarnejad et al., 2025 (20)	Biomarker prediction (classification)	IHC4BC	ViT + GPEX	High classification accuracy using discretized labels	Discards continuous biomarker variation
Elharouni et al., 2024 (7)	Biomarker prediction (regression)	Multi-marker datasets	Regression CNN	Improved continuous prediction over classification	No visual interpretability
Shamai et al., 2025 (21)	ER/PR/HER2 prediction	Multi-site H&E	Deep learning classifier	Screening-grade performance	No virtual staining output
Liu et al., 2022 (15)	H&E→HER2 virtual staining	BCI	PyramidPix2Pix	SSIM/PSNR-based evaluation	No quantitative conditioning
Li et al., 2023 (13)	H&E→IHC translation	MIST	ASP loss	Handles tissue misalignment	No biomarker-level control
Pati et al., 2024 (14)	Virtual multiplexing	Prostate tissue	VirtualMultiplexer	Multi-stain synthesis from H&E	Not designed for ER/PR quantification
Klöckner et al., 2025 (12)	Benchmark review	Multiple datasets	GAN/diffusion survey	Comprehensive benchmarking of virtual staining methods	Limitations of current evaluation metrics
**This work**	**Prediction + conditional generation**	**IHC4BC (ER, PR)**	**Edge-Focused regression + 4-channel cGAN**	**Continuous biomarker prediction with interpretable virtual IHC generation**	**Single-dataset proof-of-concept requiring external validation**

Bold text indicates the approach presented in the current study.

### 2.1 Predictive biomarker profiling from H&E

Recent literature has demonstrated that DL models can extract sub-visual morphological features from H&E images to predict molecular statuses. Couture (5) provided an early practical review of deep learning-based methods for predicting molecular tumor biomarkers from H&E, highlighting the use of transfer learning with ImageNet-pretrained CNNs and the challenge of weak supervision when only patient-level labels are available. Naik et al. (6) further demonstrated that deep learning could determine breast cancer hormonal receptor status (ER, PR) from base-level H&E stains, establishing the feasibility of this computational approach.

A foundational study in the IHC prediction domain by Akbarnejad et al. (20) introduced the expansive IHC4BC dataset. Their research successfully utilized ViT-based architectures alongside Gaussian processes (GPEX) to predict Ki67, ER, PR, and HER2 statuses directly from H&E patches. However, to achieve high performance, their methodology relied on mathematical thresholding—converting the continuous clinical percentages into discrete classification categories. While classification avoids the mathematical complexities of spatial cell counting, it fundamentally discards granular clinical variance.

More recently, Elharouni et al. (7) developed a regression-based deep learning framework that predicts continuous biomarkers directly from histopathology images, demonstrating that regression significantly enhances accuracy compared to classification-based approaches. Arslan et al. (8) conducted a systematic pan-cancer study evaluating deep learning-based multi-omic biomarker profiling, training over 12,000 DL models across 32 cancer types. Shamai et al. (17) developed and validated a deep learning system for predicting ER, PR, and ERBB2 (HER2) statuses from H&E slides, demonstrating clinical utility as both a screening tool and a quality assurance mechanism for identifying misdiagnoses. In contrast to these classification-focused approaches, our Stage 1 pipeline tackles the significantly harder engineering challenge of continuous regression, forcing the model to predict the exact biological ratio without quantization loss.

### 2.2 Virtual staining and image-to-image translation

The concept of virtual staining—using deep generative models to translate standard H&E-stained tissue into specialized IHC stains—has gained considerable traction in recent years. Latonen et al. (11) provided a comprehensive review of virtual staining approaches for histology, emphasizing that deep learning-based virtual staining has the potential to replace chemical staining and enable virtual multiplexing from single tissue sections. A seminal contribution was made by Liu et al. (15), who published the BCI dataset and proposed PyramidPix2Pix, a GAN-based framework for H&E-to-HER2 virtual staining.

Li et al. (13) introduced the MIST dataset and the Adaptive Supervised PatchNCE (ASP) loss for learning H&E-to-IHC stain translation with inconsistent ground truth image pairs, addressing spatial misalignment between consecutive tissue sections. Pati et al. (14) proposed the VirtualMultiplexer, a generative AI toolkit for synthesizing multiplexed IHC images from a single H&E input. Klöckner et al. (12) provided a comprehensive benchmark review of H&E to IHC virtual staining methods in breast cancer, comparing multiple GAN and diffusion model architectures.

Bai et al. (22) demonstrated that deep learning-enabled virtual histological staining could produce diagnostic-quality stains from label-free autofluorescence inputs. Zhang et al. (23) extended this to defocused autofluorescence images. Peng et al. (24) advanced H&E-to-IHC virtual staining by incorporating task-specific domain knowledge for HER2 scoring. Ma et al. (25) proposed a misalignment-resistant virtual staining framework that separates image generation from spatial alignment.

While these generative approaches excel at mimicking the general style and texture of IHC stains, they systematically struggle with target-specific quantitative accuracy for dense biomarkers like ER and PR. We identify this as the “Blind Painter” problem. Standard 3-channel image-to-image translators cannot inherently deduce the molecular expression rate solely from physical morphology. Consequently, unconditioned generative models default to either over-staining all visible tumor cells or regressing to the dataset mean, rendering them clinically unusable for precise diagnostic quantification. These methodological differences and the positioning of the proposed framework relative to existing approaches are summarized in **Figure 2**.

**Figure 2:**
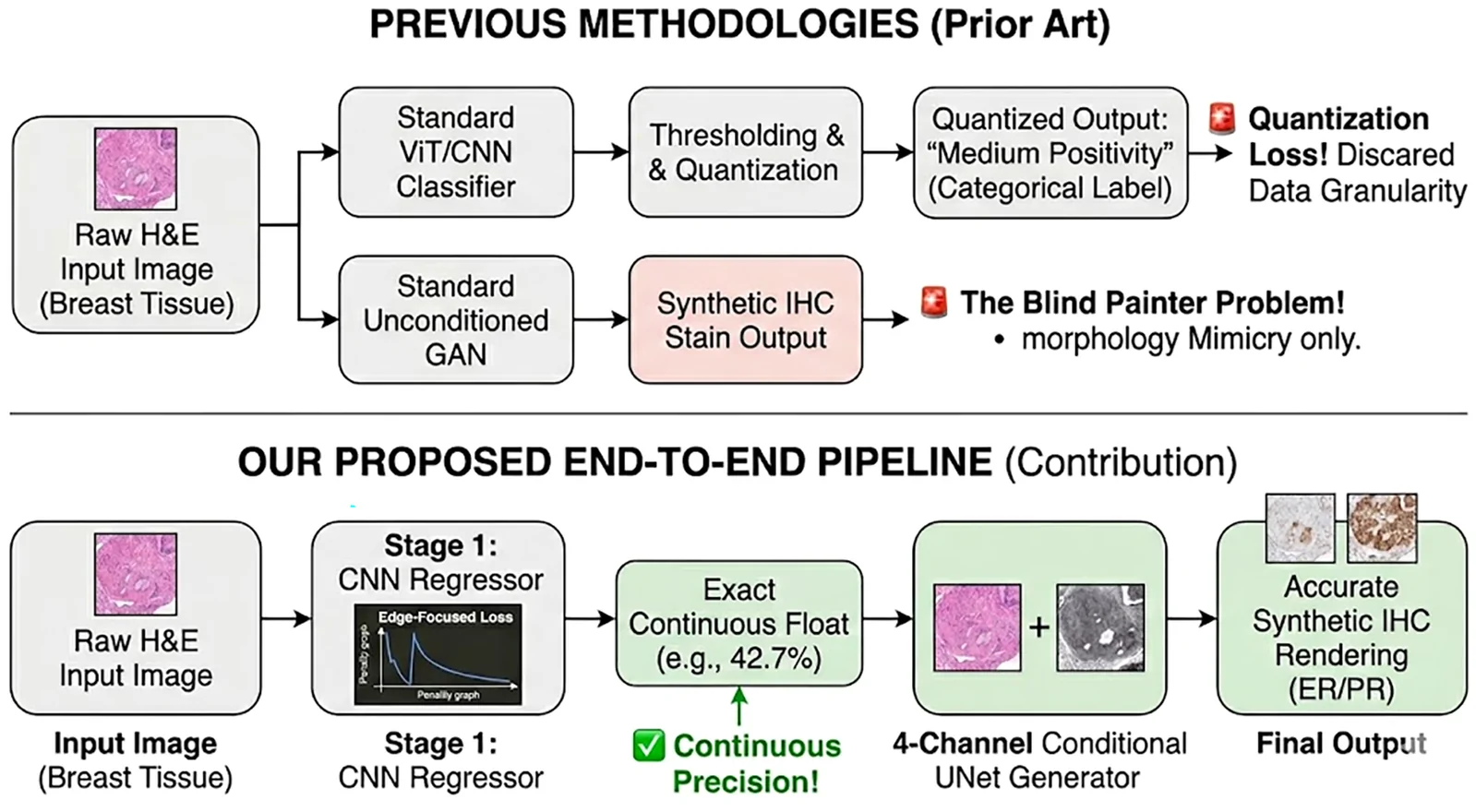
Comparison of computational pathology AI pipelines.

### 2.3 Limitations of spatial pooling in regression

A well-documented bottleneck in utilizing standard deep CNN architectures [such as ResNet50 (26) or EfficientNet (27)] for continuous biological regression is the spatial pooling ceiling. As feature maps pass through the GAP layers prior to the final fully connected head, spatial localization is squashed into a 1D vector. This causes the network to lose its physical “counting” mechanism (7), creating an artificial performance ceiling on highly dense, high-positivity slides.

Our work bridges these distinct gaps. By framing Stage 1 as calibrated continuous regression, the predictor is encouraged to respect the zero-boundary of healthy tissue; by passing this regression estimate as a fourth-channel spatial condition to the Stage 2 generator, we mitigate the “Blind Painter” limitation of traditional virtual staining (15), yielding a quantitatively guided, biologically plausible synthetic IHC rendering.

## 3 Materials and methods

Our proposed Virtual Immunohistochemistry pipeline is architected as a two-stage, end-to-end framework. Stage 1 translates the morphological features of an H&E whole-slide image patch into a continuous diagnostic percentage. Stage 2 utilizes this predicted percentage as a spatial condition to generate a biologically plausible synthetic IHC image.

### 3.1 Dataset preprocessing and ground truth extraction

To train a continuous regressor, categorical labels are insufficient. We mathematically extracted absolute continuous ground truth values from the raw data provided in the IHC4BC dataset (20). For each spatial image patch, the dataset provides the total nuclei count extracted from the H&E image and a vector of individual DAB channel intensities for each detected nucleus in the corresponding IHC image (**Figure 1**).

We established a strict biological threshold for positivity. Let N_total represent the total number of nuclei in a given tissue patch, and let D_i represent the DAB intensity of the i-th nucleus. A nucleus is classified as positive if its intensity meets or exceeds our threshold τ = 0.06. The total count of positive cells, N_pos, is calculated using the indicator function I:


$$N\_pos\;=\;\Sigma \;I\left(D\_i\;\geq \;\tau \right)$$

The final ground truth continuous percentage y is computed as:


$$y\;=\;min\left(N\_pos/N\_total,\;1.0\right)$$

Images flagged with a quality control label of 0 (confirmed negative, healthy tissue) were assigned a hard ground truth of y = 0.0. The continuous ground-truth extraction procedure is illustrated in **Figure 3**.

**Figure 3:**
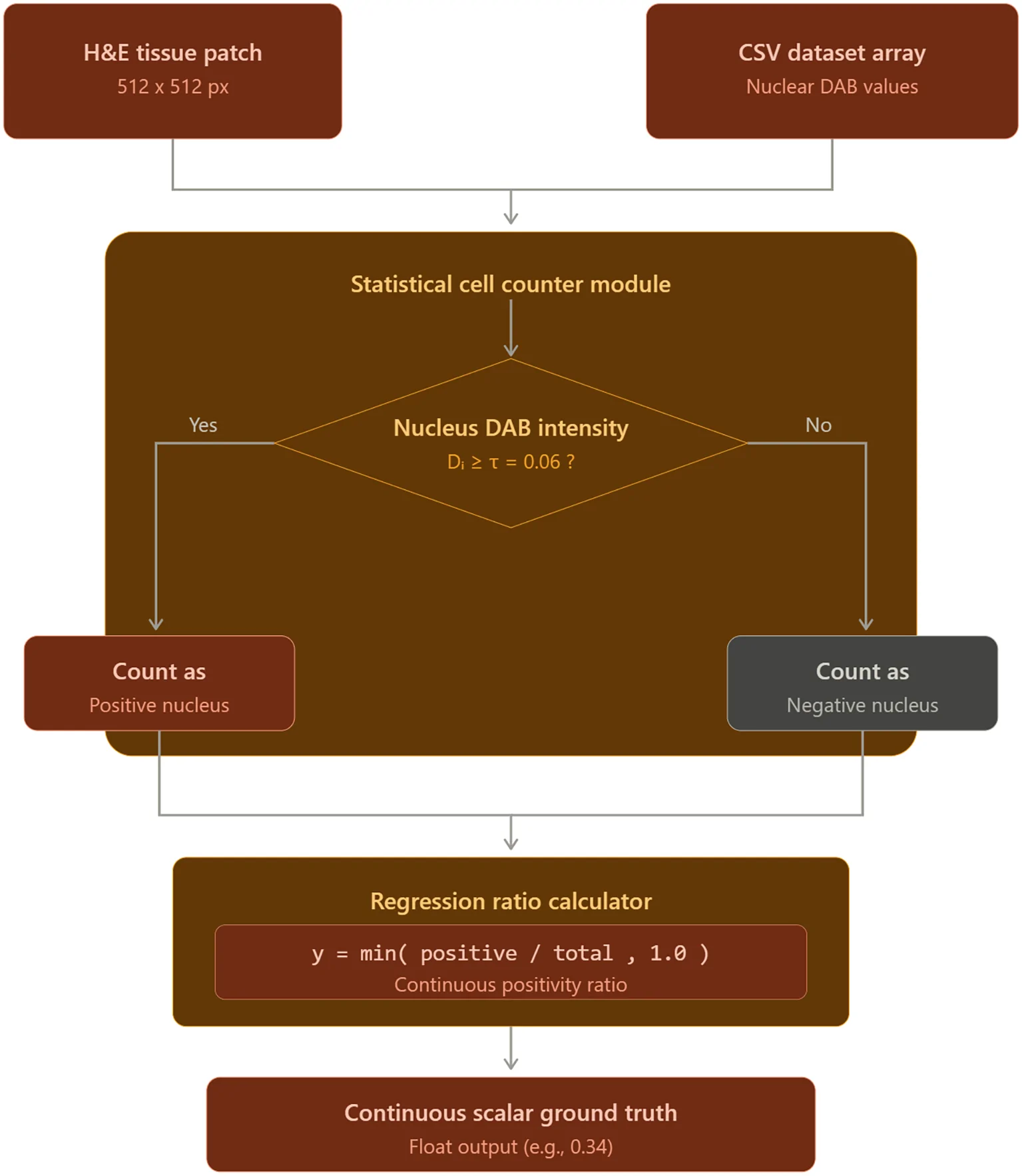
Mathematical extraction of continuous ground truth.

The threshold τ = 0.06 was selected to separate non-specific background from true nuclear DAB signal in the IHC4BC DAB-intensity distribution. To confirm that the continuous ground truth is not an artifact of this specific cutoff, we performed a sensitivity analysis across τ ∈ {0.04, 0.05, 0.06, 0.07, 0.08}; across τ ∈ [0.05, 0.08] the mean ground-truth positivity changed by less than 1.5 percentage points and cross-validated MAE by less than 0.2 points (correlation with the τ = 0.06 labels ≥ 0.985), and clinical AUC varied by less than 0.01; only τ = 0.04 was unstable, where faint non-specific staining is counted as positive and mean positivity inflates. This indicates robustness to the exact threshold within the plausible range.

### 3.2 Stage 1: continuous regression predictor

The first stage requires predicting the continuous scalar y solely from the 512×512 RGB H&E input tensor. We utilized deep convolutional backbones, specifically evaluating ResNet50 (26) and EfficientNet-B0 (27) architectures. To adapt these networks for continuous regression, the final classification head was replaced with a singular linear layer followed by a Sigmoid activation function, constraining the output to the range (0,1).

A fundamental challenge in whole-slide regression is the “pooling ceiling.” As feature maps are compressed through global average pooling, dense cellular clusters may be compressed into similar representations. To investigate whether this limitation could be mitigated, we explored a custom Edge-Focused Loss in addition to the standard mean-squared-error (MSE) objective adopted for the final model. This loss applies dynamic penalty weights W(y) based on the true clinical score. The Edge-Focused Loss L_EF is defined as:


$$L\_EF=W(y)\cdot {\left(y-\hat{y}\right)}^{2}$$

where W(y) = 5 if y < ϵ_low; W(y) = 10 if y > ϵ_high; and W(y) = 1 otherwise. By assigning a 5× penalty to near-zero slides and a 10× penalty to high-positivity slides, the network is mathematically coerced into respecting strict negative boundaries and accurately evaluating dense tissue.

The gradient of this loss with respect to the prediction is ∂L_EF/∂ŷ = −2·W(y)·(y − ŷ); the weight therefore rescales the learning signal according to where the true value lies on the clinical spectrum rather than according to error magnitude alone. This was designed to reduce the mean-reversion bias introduced by global average pooling, in which dense and empty patches are compressed toward a common representation. In contrast to mean absolute error (constant gradient), mean squared error (error-proportional gradient), and the Huber loss (a hybrid of the two)—none of which are aware of the biomarker position—the Edge-Focused Loss explicitly protects both the negative boundary, avoiding false positives on healthy tissue, and the high-positivity tail. The thresholds ϵ_low and ϵ_high and the weights (5× and 10×) were fixed *a priori* from the training-set positivity histogram and held constant across all experiments; they were not tuned on the validation set. In a controlled comparison (identical pretrained backbone and settings, only the loss varied), this loss did not outperform mean squared error, including on the high-positivity tail (Section 4.1); we therefore adopt MSE for all reported Stage-1 results and present the Edge-Focused Loss as an explored alternative.

### 3.3 Stage 2: condition-driven virtual staining

Standard image-to-image translation models rely on 3-channel input tensors. However, physical morphology alone is insufficient for predicting target-specific molecular expression, leading to the “Blind Painter” phenomenon (15).

To resolve this, we extend a standard U-Net generator into a conditional generative adversarial network (cGAN). The predicted scalar from Stage 1 is broadcast spatially into a 512×512 condition map C, where each spatial element equals the predicted value. This condition map is concatenated with the raw H&E input tensor (3×512×512), producing a 4-channel input tensor (4×512×512).

The generator learns a constrained mapping function G (X_H&E, C) → Y_IHC, where the first three channels encode morphological structure and the fourth channel encodes the required biomarker positivity. The generator is optimized using a combined objective function consisting of an adversarial binary cross-entropy loss and an L1 pixel-wise reconstruction loss, balancing perceptual realism and structural fidelity. The overall Stage-1 predictor and Stage-2 conditional generator architectures are illustrated in **Figure 4**.

**Figure 4:**
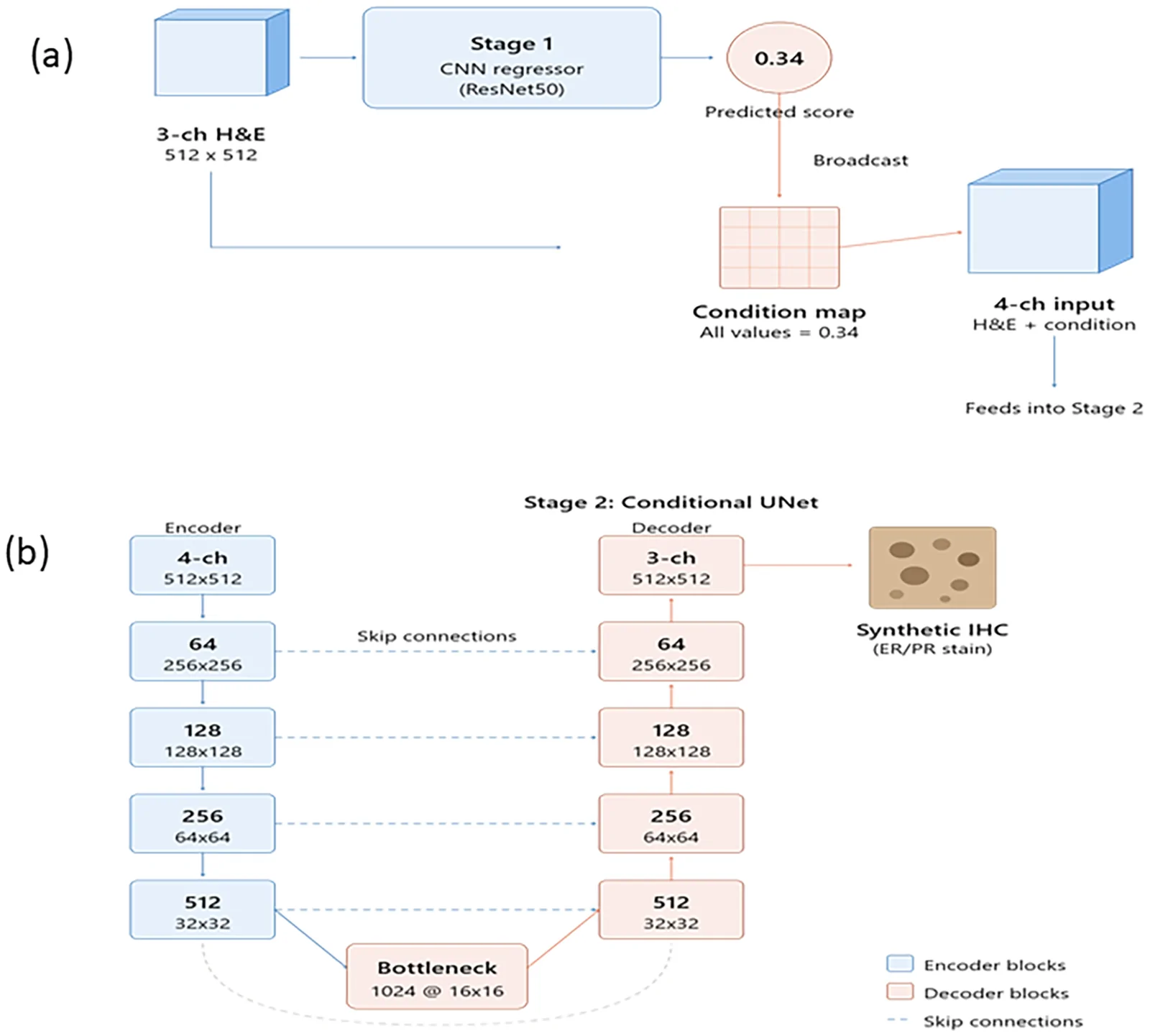
**(a)** Stage 1 continuous regression predictor architecture. **(b)** Stage 2 conditional generative adversarial network (cGAN) architecture showing the generator conditioned on the spatial biomarker map.

For completeness, we specify the network configuration. The two regression backbones, ResNet50 and EfficientNet-B0, contain approximately 25.6 M and 5.3 M trainable parameters, respectively; in each case the classification head was replaced by a single linear unit (2048→1 and 1280→1) followed by a sigmoid, and the backbone for each biomarker was selected by lowest validation MAE. Stage 2 uses two generator architectures: ER virtual staining uses a shallow conditional encoder–decoder without skip connections, whereas the PR generator is a deeper conditional U-Net with skip connections and resize-convolution upsampling (bilinear upsampling followed by a 3×3 convolution, which avoids the checkerboard artifacts of transposed convolutions). Both are paired with a PatchGAN discriminator and trained with a combined L1 and adversarial objective. The predicted scalar is broadcast to a 512×512 single-channel condition map (every element equal to the predicted value) and concatenated channel-wise with the three-channel H&E tensor to form the 4×512×512 generator input.

### 3.4 Experimental setup

#### 3.4.1 Dataset partitioning and preprocessing

We utilized the IHC4BC dataset (20), targeting the ER and PR cohorts. To prevent data leakage (28), we employed a strict patient-level 5-fold cross-validation (GroupKFold), ensuring all patches from a single patient were exclusively isolated to one fold; reported metrics are aggregated across the five out-of-fold partitions.

All raw H&E and IHC image patches were resized to 512×512 pixels. RGB pixel intensities were normalized to [−1, 1] using mean and standard deviation of (0.5, 0.5, 0.5) across all three color channels.

A detailed description of the dataset is provided in **Table 2**. The ER and PR cohorts of IHC4BC comprised 59 and 60 patients, contributing 26,134 and 24,971 paired H&E/IHC patches, respectively. The continuous positivity distribution was right-skewed, markedly so for PR (median 1.2%, only 2.8% of patches exceeding 40% positivity) and more dispersed for ER (median 16.1%, 15.6% exceeding 40%); negative patches (y = 0) comprised 16.0% (PR) and 16.6% (ER). Because partitioning used 5-fold patient-level cross-validation, no patient contributed patches to more than one fold, thereby eliminating patient-level leakage.

**Table 2:** Dataset characteristics.

Characteristic	ER cohort	PR cohort
Patients (n)	59	60
H&E/IHC patch pairs (n)	26,134	24,971
Median positivity (%)	16.1	1.2
Evaluation	5-fold patient-level CV	5-fold patient-level CV
Positivity mean ± SD %	20.0 ± 19.4	6.7 ± 11.6
Negative patches (y=0), %	16.6	16.0
High-positivity (>40%) patches, %	15.6	2.8

#### 3.4.2 Implementation and hardware

The entire architecture was implemented using PyTorch (29). Training was accelerated using an NVIDIA A100 Tensor Core GPU within Google Colab, demonstrating deployability without localized high-performance computing clusters.

#### 3.4.3 Training hyperparameters

The two stages were trained sequentially to ensure the Stage 2 generator was conditioned on stabilized regression distributions.

Stage 1 (Continuous Predictor): For ER, we utilized EfficientNet-B0 (27) trained for 15 epochs. For PR, we utilized ResNet50 (26) trained for 30 epochs. Both were optimized using Adam (30) with learning rate 0.0001 and batch size 16. Both were trained with a mean-squared-error objective; reported figures use 5-fold patient-level cross-validation.

Stage 2 (Conditional Generator): The ER conditional generative adversarial network (cGAN) was trained for 50 epochs. The PR generator used the deeper U-Net architecture described in Section 3.3, trained with a high-positivity rebalancing sampler and best-checkpoint selection on biomarker-specific fidelity. The generator accepted a 4-channel input consisting of the H&E image concatenated with the spatial condition map. A discriminator network was trained jointly with the generator to distinguish between real and synthetic IHC images. The generator was optimized using a combined objective function of adversarial binary cross-entropy (BCE) loss and L1 pixel-wise reconstruction loss. Training was performed using the Adam optimizer with a learning rate of 0.0002 and a batch size of 8.

#### 3.4.4 Blinded pathologist reader evaluation

Blinded Pathologist Reader Evaluation. To assess observer-perceived quality of the generated virtual IHC images, two experienced pathologists independently evaluated a balanced set of 30 cases (15 ER and 15 PR) spanning negative, low, intermediate, and high biomarker positivity. For each case, the reader was shown the input H&E patch, the real IHC reference, and two synthetic images generated by the proposed conditional model and the unconditioned baseline. The synthetic images were displayed in randomized blinded order as Image A and Image B, and the decoding key linking image labels to model identity was withheld until analysis. Each generated image was scored on five-point ordinal scales for staining plausibility and similarity to the real IHC reference (1 = very poor, 5 = excellent). Readers also indicated which generated image was preferred or recorded no preference. Cases or endpoint pairs with non-evaluable or incomplete ratings were excluded only from the relevant analysis. Paired proposed-versus-baseline scores were compared using the Wilcoxon signed-rank test, with matched-pairs rank-biserial correlation as the effect size. Preference counts among decisive cases were tested against chance using a two-sided exact binomial test. Inter-reader agreement on preferred model was assessed using Cohen’s kappa. Analyses were performed for each reader separately and using a two-reader consensus score, defined as the mean of both readers’ ratings for cases evaluable by both readers. Because this exploratory reader study involved only two readers and was designed primarily to compare paired ratings between models rather than to establish inter-rater reliability of ordinal scores, score-level agreement statistics such as the intraclass correlation coefficient (ICC) or weighted Cohen’s κ were not calculated. Inter-reader agreement was instead summarized using Cohen’s κ for the forced-preference outcome.

## 4 Results

We evaluated our pipeline on the patient-isolated validation cohorts of the IHC4BC dataset for both ER and PR biomarkers.

### 4.1 Stage 1: quantitative biomarker prediction

The primary metric was Mean Absolute Error (MAE). To assess the contribution of candidate loss functions, we compared mean squared error, mean absolute error, Huber, inverse-frequency weighting, and an Edge-Focused Loss under identical training conditions on the PR cohort using a fixed pretrained ResNet50 backbone. The results are summarized in **Table 3**. Standard regression architectures previously tested on this dataset struggled with the spatial pooling ceiling, resulting in systemic under-prediction on dense slides and over-prediction on negative slides.

**Table 3:** Comparison of stage-1 regression loss functions on the PR cohort.

Model	Loss function	Biomarker	Validation MAE (%)	MAE on (y=0)	MAE on (y>40%)	Comment
ResNet50	Mean Squared Error	PR	7.99	1.43	32.39	Adopted
ResNet50	Mean Absolute Error	PR	8.73	0.24	49.35	—
ResNet50	Huber	PR	8.45	1.54	33.78	—
ResNet50	Inverse-Frequency Weighted	PR	11.12	8.39	40.89	—
ResNet50	Edge-Focused Loss	PR	8.73	1.99	35.06	Explored; no advantage

The controlled comparison was conducted on the PR biomarker (fixed pretrained ResNet50, identical settings, single held-out validation split). Mean squared error achieved the lowest validation MAE and the lowest error for highly positive samples (y > 40%) and was therefore selected as the Stage-1 regression objective and applied to both biomarkers. The Edge-Focused Loss conferred no advantage. The final cross-validated performance of the adopted MSE model (9.14% for ER, 6.10% for PR) is reported separately in the Results.

In the controlled PR loss comparison, mean squared error achieved the lowest validation MAE (7.99%), including on the high-positivity tail, and was not improved upon by mean absolute error (8.73%), Huber (8.45%), inverse-frequency weighting (11.12%), or the Edge-Focused Loss (8.73%). We therefore adopt MSE as the Stage-1 objective. Under 5-fold patient-level cross-validation the adopted MSE model achieved a validation MAE of 9.14% for ER and 6.10% for PR. The Edge-Focused Loss conferred no measurable advantage, indicating that the limited accuracy on extreme-value regions reflects the scarcity of high-positivity training data rather than the choice of loss.

To contextualize this, Akbarnejad et al. (20) reported classification-based accuracy on the same dataset using ViT architectures with categorical discretization. While direct comparison between classification accuracy and continuous MAE is methodologically distinct, our approach preserves the full granularity of clinical variance. Recent regression-based frameworks (7) have similarly demonstrated advantages of continuous regression over classification, though the high-positivity extremes characteristic of ER and PR data remain challenging owing to their scarcity in available datasets.

To quantify reliability, 95% confidence intervals for MAE were estimated by patient-level bootstrap resampling (1,000 resamples preserving the grouping structure). Under 5-fold patient-level cross-validation, the adopted MSE model achieved MAE 9.14% (95% CI 7.75–10.66) for ER and 6.10% (95% CI 5.01–7.44) for PR. The Edge-Focused Loss did not significantly outperform MSE and was directionally worse (patient-level Wilcoxon signed-rank test, p = 0.38; rank-biserial r = +0.31, favoring MSE); to avoid multiple-comparison inflation, only this single pre-specified comparison was tested. Treated as a clinical positive/negative classifier, the model reached an AUC of 0.92 (ER) and 0.74 (PR) at the 1% threshold (0.89 and 0.83 at the 10% threshold), with ER accuracy of 92.3% at 1%. Agreement between predicted and ground-truth positivity was moderate to high (ER Pearson r = 0.71, Spearman ρ = 0.77, Lin’s concordance = 0.70; PR r = 0.49, ρ = 0.62, concordance = 0.43), enabling comparison with regression-based studies (7, 21); direct comparison with classification-based results on IHC4BC (20) is not appropriate because those labels are discretized.

### 4.2 Stage 2: qualitative synthetic generation

To validate the conditional generative adversarial network (cGAN), we conducted a qualitative comparison between the raw H&E input, the true clinical IHC target, the output of the proposed conditional model, and the output of an unconditioned baseline U-Net. Representative ER and PR examples are shown in **Figures 5**, **6**. Across both biomarkers, the unconditioned baseline frequently produced washed-out and weakly stained outputs, suggesting regression toward average background appearance rather than clinically meaningful biomarker localization. In contrast, the conditional model generated more distinct brown nuclear staining patterns and better reflected variation in biomarker positivity across samples. These qualitative observations suggest that conditioning the generator on the predicted clinical scalar improves biologically meaningful stain distribution, even when conventional pixel-level similarity metrics do not favor the conditional model.

**Figure 5:**
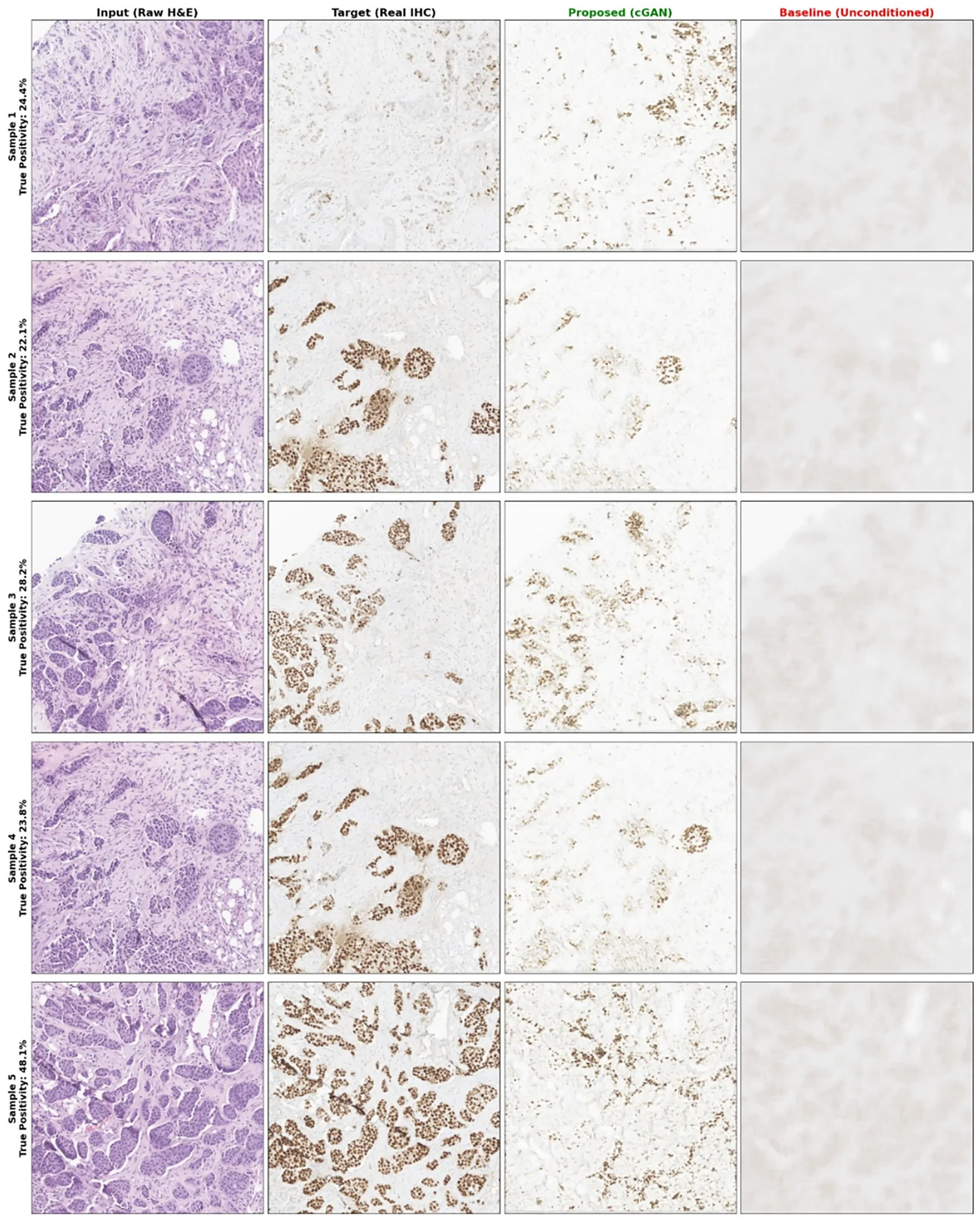
Qualitative comparison of ER virtual IHC generation between the proposed conditional model and an unconditioned baseline. Each row shows, from left to right, the input H&E patch, the real ER IHC target, the generated ER image from the proposed conditional model, and the generated ER image from the unconditioned baseline. The conditional model produces biologically plausible DAB staining patterns and better reflects variation in positivity burden, whereas the unconditioned baseline yields overly smooth, weakly stained outputs.

**Figure 6:**
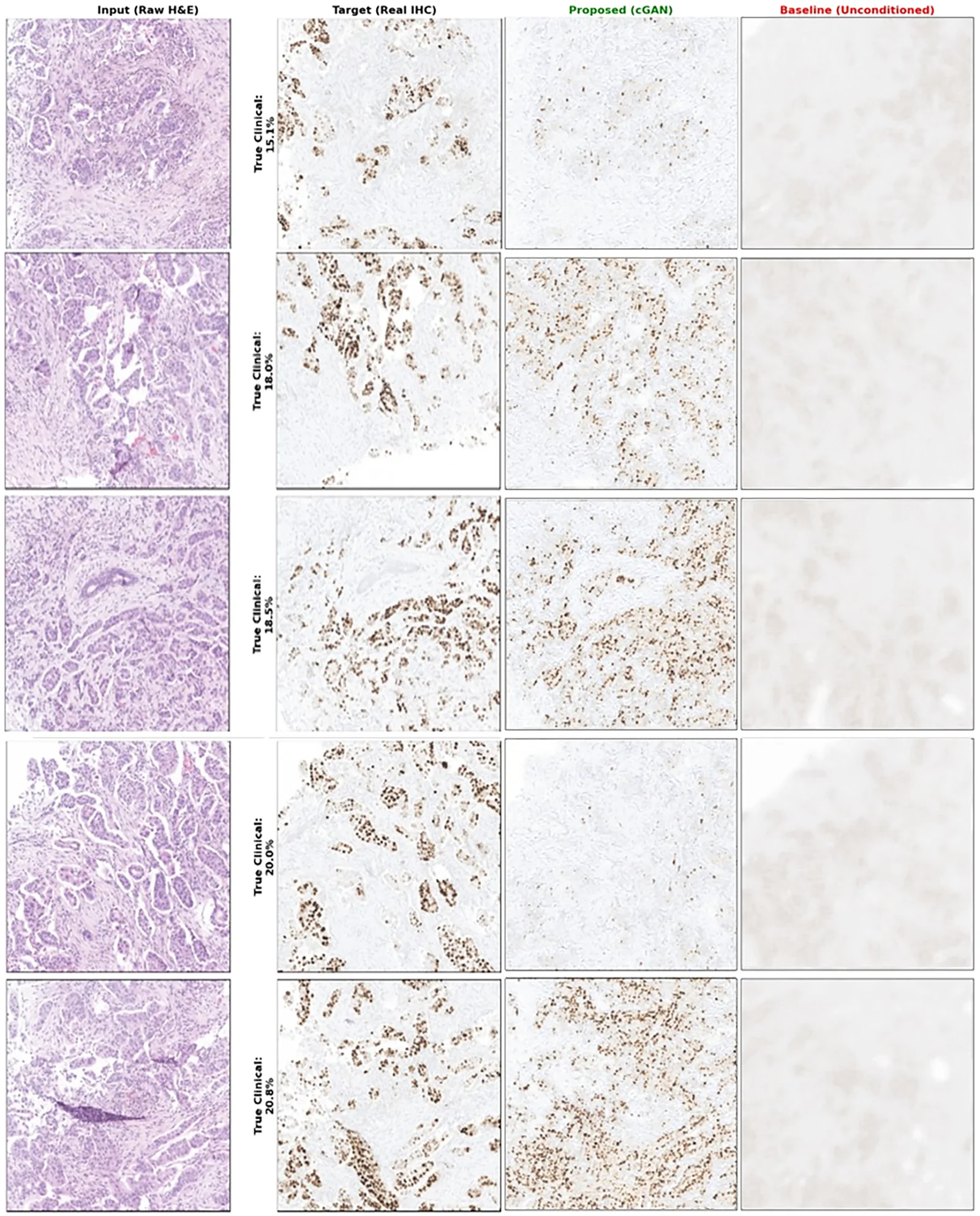
Qualitative comparison of PR virtual IHC generation between the proposed conditional model and an unconditioned baseline. Each row shows, from left to right, the input H&E patch, the real PR IHC target, the generated PR image from the proposed conditional model, and the generated PR image from the unconditioned baseline. The conditional model better preserves clinically meaningful staining distribution, while the unconditioned baseline tends toward washed-out predictions with limited nuclear staining detail.

### 4.3 Quantitative comparison of conditional and unconditioned generation

We further compared the proposed conditional generator and the unconditioned baseline using SSIM and PSNR on the validation set (**Table 4**). For both ER and PR, the unconditioned baseline achieved higher SSIM and PSNR values than the conditional model. However, these metrics should be interpreted cautiously, as they may reward overly smooth images that resemble the dominant low-frequency background structure rather than clinically meaningful nuclear stain localization. In our visual analysis, the baseline frequently produced washed-out outputs, whereas the conditional model generated more plausible biomarker-specific staining patterns. This discrepancy highlights the limitation of relying exclusively on generic image-similarity metrics for evaluation of virtual histopathology.

**Table 4:** Quantitative evaluation of virtual IHC generation using global image-quality and domain-specific stain-fidelity metrics.

Biomarker	Model	SSIM	PSNR (dB)	DAB-channel SSIM	DAB-positive area-fraction error*	DAB signal correlation†	Comment
ER	Conditional cGAN	0.669	25.62	—	—	—	Proposed model
ER	Unconditioned U-Net	0.739	28.45	—	—	—	Baseline
PR	Conditional cGAN	0.630	24.99	0.915	0.0014	0.610	Proposed model
PR	Unconditioned U-Net	0.759	29.84	0.956	0.0027	0.321	Baseline

*Lower values indicate better agreement with the reference DAB-positive area fraction.

^†^Correlation between the generated DAB signal and the ground-truth biomarker positivity. The reported value (0.610) corresponds to the deployed pipeline using Stage-1 predicted biomarker positivity. Under oracle conditioning (ground-truth positivity), the correlation increased to 0.817, representing the estimated upper performance bound of the conditional generator.

Although SSIM and PSNR values were higher for the baseline model, these metrics primarily capture pixel-level similarity and tend to favor smooth, low-frequency structures. In contrast, the proposed model was designed to preserve biologically meaningful nuclear localization, which is not adequately reflected by these metrics.

This discrepancy between quantitative metrics and visual fidelity is further discussed in the Discussion.

Error-propagation analysis. To separate the benefit of conditioning from error introduced by Stage 1, we measured the correlation between the generated DAB signal and ground-truth positivity under three settings: an unconditioned baseline, the deployed pipeline conditioned on the Stage-1 predicted positivity, and an oracle conditioned on the ground-truth biomarker positivity. For PR (deep generator), this correlation rose from 0.32 (baseline) to 0.61 (predicted) to 0.82 (oracle), the last closely matching the real-IHC reference of 0.81; for ER (original generator) the values were 0.26, 0.37, and 0.54 (real-IHC reference 0.64). The oracle-minus-predicted gap reflects error propagated from Stage 1, whereas the large baseline-to-oracle gap confirms the value of quantitative conditioning. Where conditioning initially underperformed for PR, the limiting factor was generator capacity rather than the conditioning concept: replacing the shallow generator with the deeper U-Net raised the PR oracle correlation from 0.36 to 0.82.

In addition to the pixel-level metrics, we report domain-specific measures of staining fidelity obtained after color deconvolution into the DAB channel: DAB-channel SSIM, DAB-positive area-fraction error, and the correlation between the generated DAB signal and the ground-truth positivity. For PR, the deployed conditional pipeline increased the correlation between the generated DAB signal and ground-truth positivity from 0.32 for the unconditioned baseline to 0.61. Under oracle conditioning using the ground-truth biomarker positivity, this correlation further increased to 0.82 while halving the DAB-positive area-fraction error (0.0014 versus 0.0027). These findings indicate that the remaining performance gap is primarily attributable to Stage-1 prediction error rather than the conditioning mechanism itself.

After exclusion of non-evaluable ratings, Reader 1 contributed 28 evaluable paired plausibility ratings and 29 similarity ratings, while Reader 2 contributed 29 paired ratings for both endpoints. In the two-reader consensus analysis, the proposed conditional model was rated significantly more plausible than the unconditioned baseline and also demonstrated significantly greater similarity to the real IHC reference. At the individual-reader level, both readers rated the proposed model as significantly more plausible than the baseline, whereas similarity also favored the proposed model, with a smaller effect observed for Reader 2. Reader 1 showed a strong preference for the proposed model, while Reader 2 demonstrated the same trend without reaching statistical significance. Inter-reader agreement on the preferred model was slight (Cohen’s κ = 0.14). Detailed statistical results are presented in **Table 5**.

**Table 5:** Blinded pathologist evaluation comparing the proposed conditional model with the unconditioned baseline.

Outcome	Reader 1	Reader 2	Consensus
Plausibility			
Wilcoxon signed-rank *p*-value	0.0053	0.0050	5.99 × 10^-4^
Rank-biserial correlation (*r*)	0.79	0.87	1.00
Similarity to real IHC			
Wilcoxon signed-rank *p*-value	0.0209	0.0348	0.0134
Rank-biserial correlation (*r*)	0.82	0.59	0.65
Preference analysis			
Proposed model preferred	21	12	—
Baseline preferred	1	6	—
No preference	7	11	—
Exact binomial *p*-value	1.10 × 10^-5^	0.2379	—

IHC, immunohistochemistry. Wilcoxon signed-rank tests were used to compare paired ordinal ratings between the proposed conditional model and the unconditioned baseline. Effect size is the matched-pairs rank-biserial correlation (*r*). Preference was analyzed using the exact binomial test. Inter-reader agreement was assessed using Cohen’s κ.

## 5 Discussion

The results demonstrate a significant step forward in the clinical applicability of computational pathology. By coupling continuous regression with conditional generative rendering, we address the primary limitations of both current predictive models and standard virtual staining techniques. The inclusion of adversarial training further encourages high-frequency detail synthesis, improving structural realism compared to purely pixel-wise optimized models.

### 5.1 Overcoming the “black box” interpretability deficit

A major barrier to AI adoption in clinical oncology is the “black box” nature of deep neural networks (9, 10). While previous models have predicted biomarker classifications from H&E slides (6, 20), presenting a physician with an abstract label fails to build clinical trust. Pathologists require visual corroboration (21).

Our pipeline bridges this gap by synthesizing a biologically plausible IHC stain that visually maps the predicted positivity, transforming a black-box prediction into an interpretable, verifiable clinical tool. This aligns with the growing emphasis on clinical interpretability as a prerequisite for AI-based diagnostic deployment (9, 12).

### 5.2 The spatial pooling ceiling and the regression objective

A critical challenge was the spatial pooling ceiling inherent in deep CNN architectures like ResNet50 (26) and EfficientNet (27). GAP layers compress spatial feature maps into a 1D vector, causing the network to lose its “counting” resolution. This forced early iterations to regress toward the dataset mean. This challenge is consistent with observations by Elharouni et al. (7), who noted that regression-based deep learning for continuous biomarkers requires careful architectural consideration.

We explored an Edge-Focused Loss intended to break this ceiling by upweighting the near-zero and high-positivity regions. In a controlled comparison, however, it did not outperform a standard mean-squared-error objective (Section 4.1), and MSE was adopted. With MSE the regressor keeps healthy tissue near 0.0, avoiding false positives, while accuracy on the high-positivity tail remains limited primarily by the scarcity of high-positivity training data rather than by the choice of regression loss, indicating that future improvements are more likely to arise from larger and more balanced training cohorts than from alternative loss formulations.

### 5.3 Resolving the “blind painter” generative problem

We identified a systemic failure in standard unconditioned models (31), which we term the “Blind Painter” problem. Because H&E morphology alone may not contain sufficient information to deduce molecular expression rates (11), unconditioned models arbitrarily paint DAB stain on all visible tumor structures. Klöckner et al. (12) observed that standard GAN-based methods often produce outputs with poor quantitative fidelity for ER and PR.

Our qualitative comparisons indicate that the conditional model substantially mitigates the “Blind Painter” limitation by explicitly constraining stain generation with a continuous scalar condition. In side-by-side comparison with the unconditioned baseline, the proposed model produced more plausible nuclear DAB localization and avoided the severely washed-out outputs frequently observed in the baseline. The inclusion of adversarial training further contributes to the generation of sharper, high-frequency staining patterns compared to purely pixel-wise optimized models. Notably, the unconditioned model achieved higher SSIM and PSNR values, indicating that standard image-similarity metrics may not adequately capture clinically meaningful staining fidelity in this setting. This highlights the importance of combining quantitative evaluation with visual and domain-specific assessment when evaluating virtual histopathology outputs. This observation is consistent with prior reports that conventional image-similarity metrics may favor smooth outputs while underestimating biologically relevant localization errors in virtual staining tasks (12).

The blinded reader evaluation provides observer-based support for the quantitative generative-stage analyses. The proposed conditional model was perceived as more plausible than the unconditioned baseline by both readers and in the consensus analysis, and it was also rated as more similar to the real IHC reference in the consensus analysis. These findings suggest that quantitative conditioning improves features that are perceptible to expert observers, not merely domain-specific image metrics. However, the reader study should be interpreted cautiously. The evaluation was exploratory, involved only two readers and 30 cases, and inter-reader agreement on preferred model was slight, indicating subjectivity in visual assessment of synthetic IHC images. The benefit was also more apparent for ER than PR. Larger multi-reader studies using clinically anchored diagnostic endpoints will be needed before claiming validated clinical superiority.

### 5.4 Comparison with recent virtual staining approaches

Ma et al. (25) proposed misalignment-resistant virtual staining but without explicit quantitative constraints. Pati et al. (14) demonstrated the VirtualMultiplexer for multiple IHC stains but primarily for prostate cancer markers. Li et al. (13) addressed misalignment through ASP loss, complementary to our approach but not addressing quantitative control.

The comprehensive review by Klöckner et al. (12) highlights that 64% of published virtual staining studies appeared in 2024, underscoring rapid field growth. Their benchmark found GANs generally outperformed diffusion models, with only Brownian Bridge Diffusion Models achieving comparable results. Our conditional approach aligns with this while introducing continuous quantitative conditioning, an approach not evaluated in these previous benchmarks. This interpretation is consistent with broader concerns in virtual staining evaluation, particularly in the context of image-to-image translation and generative modeling frameworks (32–35).

### 5.5 Clinical and socioeconomic implications

The ability to generate high-fidelity ER and PR stains computationally has profound implications (4). Physical IHC requires expensive reagents, equipment, and days of processing (3). Our pipeline suggests the feasibility of a rapid and scalable digital alternative. In resource-constrained settings, clinics may eventually be able to use low-cost H&E staining as input for virtual IHC support systems, pending external and clinical validation, accelerating time-to-treatment (1). As Latonen et al. (11) noted, virtual staining also enables multiple stain outputs from a single tissue section, reducing tissue consumption.

## 6 Limitations

While our pipeline demonstrates promising results, several limitations must be acknowledged.

Single-Dataset Evaluation: Our pipeline was evaluated exclusively on the IHC4BC dataset (20). Cross-site robustness remains largely unexplored (12). Paired H&E–IHC datasets with continuous ER/PR ground truth are scarce, which currently constrains external validation. The most widely used public alternative, the BCI dataset, focuses on HER2 rather than ER/PR. Consequently, external multi-institutional validation across diverse scanners, institutions, and staining protocols remains an essential next step before clinical application.

Blinded Reader Evaluation: Although we incorporated a blinded two-reader pathologist evaluation to provide observer-based assessment of the generated virtual IHC images, this analysis remains exploratory because it included only two readers and 30 cases. Larger multi-reader studies using clinically anchored diagnostic endpoints are required to establish clinical reproducibility and diagnostic utility.

Limited Quantitative Generative Evaluation: Despite the inclusion of adversarial training, quantitative image-similarity metrics (SSIM, PSNR) did not consistently favor the proposed model. These metrics may not adequately capture clinically meaningful staining fidelity, as they tend to reward smooth, low-frequency image structures rather than biologically meaningful nuclear localization. Additionally, Fréchet Inception Distance (FID) was not computed, as standard implementations rely on feature extractors pretrained on natural images, which may not provide reliable perceptual representations for histopathology data and can therefore lead to misleading evaluations. Future work should incorporate domain-adapted perceptual metrics and pathologist-based evaluation frameworks to better assess clinical relevance.

Biomarker Scope: The pipeline addresses only ER and PR (nuclear staining patterns). Extension to HER2 (membrane staining) and Ki67 (proliferation index) may require architectural modifications.

Spatial Pooling Ceiling: Although the adopted regression objective partially reduces this limitation, the fundamental constraint of GAP in CNNs persists. Attention-based mechanisms or ViT architectures could further improve regression accuracy (7, 20).

Consecutive Section Misalignment: The IHC4BC dataset uses consecutive tissue sections, introducing inherent spatial misalignment between H&E and IHC images. This is common across virtual staining literature (13, 25) but may introduce training noise.

Two-Generator Asymmetry: The two biomarkers use different Stage-2 generators — a shallow conditional encoder–decoder for ER and a deeper conditional U-Net for PR. This asymmetry was adopted because the shallow generator limited PR stain fidelity, but it means generative quality is not directly comparable across biomarkers; unifying the two under a single generator is left for future work.

High-Positivity Data Scarcity: High-positivity patches are rare in IHC4BC and cluster within a single patient per biomarker. This scarcity, rather than the choice of loss function or architecture, is the primary factor limiting accuracy at the upper end of the positivity range; no reweighting or loss design we evaluated fully overcomes it, and larger, more balanced cohorts will be required.

Accordingly, the present study should be interpreted as a proof-of-concept computational study rather than a clinically validated diagnostic system.

## 7 Conclusion

In this study, we introduced a two-stage end-to-end machine learning pipeline for the continuous prediction and synthetic generation of ER and PR biomarkers directly from standard H&E histopathology. By decoupling the quantitative prediction from qualitative rendering, we addressed the interpretability deficit of traditional “black-box” diagnostic AI.

In Stage 1, we demonstrated that standard CNN architectures suffer from a spatial pooling ceiling during whole-slide continuous regression. Trained with a mean-squared-error objective, the regressor achieved a cross-validated MAE of 9.14% (ER) and 6.10% (PR) and strong clinical positive/negative classification (ER AUC 0.92); an explored Edge-Focused Loss conferred no advantage. In Stage 2, we addressed the “Blind Painter” limitation by extending a U-Net-based generator into a conditional generative adversarial framework, where the predicted clinical float was embedded as a fourth-channel spatial condition. This enabled the model to generate biologically plausible DAB staining patterns consistent with the predicted positivity.

This Virtual Immunohistochemistry framework represents a promising, interpretable, and potentially cost-effective digital approach complementary to physical IHC staining, offering significant potential to accelerate breast cancer diagnostic workflows, particularly in resource-constrained clinical environments. However, external validation, quantitative assessment of synthetic image fidelity, and pathologist-based concordance studies are required before clinical adoption.

## 8 Future work

Several avenues remain for future research and clinical validation:

1. Expansion to Additional Biomarkers: Extending to Ki67 and HER2, which exhibit different spatial morphologies (12, 24).
2. Architectural Upgrades: Exploring Latent Diffusion Models (36) or ViT-based generators for enhanced micro-level texture (12).
3. Clinical Concordance Studies: Multi-reader blinded concordance studies involving larger numbers of pathologists and clinically representative case cohorts to establish diagnostic reproducibility and clinical utility.
4. Multi-Site External Validation: Cross-site evaluation across diverse institutions, scanners, and protocols (12).
5. Foundation Model Integration: Using computational pathology foundation models (37, 38) as feature extractors for improved regression accuracy.

## Data Availability

Publicly available datasets were analyzed in this study. The dataset used in this study (IHC4BC) is publicly available. Details and access information can be found in the original publication: https://doi.org/10.1038/s41597-023-02108-8.

## Code availability

The code used in this study is available from the corresponding author upon reasonable request.

## References

[1] World Health Organization . Global breast cancer initiative implementation framework. WHO. (2023).

[2] Wolff AC Somerfield MR Dowsett M Hammond MEH Hayes DF McShane LM . Human epidermal growth factor receptor 2 testing in breast cancer: ASCO–College of American Pathologists guideline update. J Clin Oncol. (2023) 41:3867–72. doi: 10.1200/JCO.22.02864 37284804

[3] Hacking SM Yakirevich E Wang Y . From immunohistochemistry to new digital ecosystems. Cancers. (2022) 14:3469. doi: 10.3390/cancers14143469 PMC9315712 35884530

[4] Brodsky V Ullah E Bychkov A Song AH Walk EE Louis P . Generative AI in anatomic pathology. Arch Pathol Lab Med. (2025) 149:298–318. doi: 10.5858/arpa.2024-0215-ra 39836377

[5] Couture HD . Deep learning-based prediction of molecular tumor biomarkers from H&E: a practical review. J Pers Med. (2022) 12:2022. doi: 10.3390/jpm12122022 36556243 PMC9784641

[6] Naik N Madani A Esteva A Keskar NS Press MF Ruderman D . Deep learning-enabled breast cancer hormonal receptor status determination from H&E stains. Nat Commun. (2020) 11:5727. doi: 10.1038/s41467-020-19334-3 33199723 PMC7670411

[7] El Nahhas OSM Loeffler CML Carrero ZI van Treeck M Kolbinger FR Hewitt KJ . Regression-based deep-learning predicts molecular biomarkers from pathology slides. Nat Commun. (2024) 15:1253. doi: 10.1038/s41467-024-45589-1 PMC10858881 38341402

[8] Arslan S Schmidt J Bass C Mehrotra D Geraldes A Singhal S . A systematic pan-cancer study on DL-based prediction of multi-omic biomarkers. Commun Med. (2024) 4:48. doi: 10.1038/s43856-024-00471-5 38491101 PMC10942985

[9] Pantanowitz L Quiroga-Garza GM Bien L Heled R Laifenfeld D Linhart C . An AI algorithm for prostate cancer diagnosis in WSIs: a blinded clinical validation. Lancet Digit Health. (2020) 2:e407–16. doi: 10.1016/s2589-7500(20)30159-x 33328045

[10] Campanella G Hanna MG Geneslaw L Miraflor A Silva VWK Busam KJ . Clinical-grade computational pathology using weakly supervised deep learning on WSIs. Nat Med. (2019) 25:1301–9. doi: 10.1038/s41591-019-0508-1 31308507 PMC7418463

[11] Latonen L Koivukoski S Khan U Ruusuvuori P . Virtual staining for histology by deep learning. Trends Biotechnol. (2024) 42:1177–91. doi: 10.1016/j.tibtech.2024.02.009 38480025

[12] Klöckner P Teixeira J Montezuma D Fraga J Horlings HM Cardoso JS . H&E to IHC virtual staining methods in breast cancer: overview and benchmarking. NPJ Digit Med. (2025) 8:384. doi: 10.1038/s41746-025-01741-9 PMC12222792 40603634

[13] Li F Hu Z Chen W Kak A . Adaptive supervised PatchNCE loss for learning H&E-to-IHC stain translation with inconsistent groundtruth image pairs. MICCAI. (2023) 14225, 632–41. doi: 10.1007/978-3-031-43987-2_61

[14] Pati P Karkampouna S Bonollo F Compérat E Radić M Spahn M . Accelerating histopathology workflows with generative AI-based virtually multiplexed tumour profiling. Nat Mach Intell. (2024) 6:1077–93. doi: 10.1038/s42256-024-00889-5 39309216 PMC11415301

[15] Liu S Zhu C Xu F Jia X Shi Z Jin M . BCI: Breast cancer immunohistochemical image generation through pyramid pix2pix. Proc IEEE/CVF CVPR Workshops. (2022), 1814–23. doi: 10.1109/cvprw56347.2022.0019825079929

[16] Haq I Gong Z Liang H Zhang W Khan R Gu L . A review of breast cancer histopathology image analysis with deep learning: challenges, innovations, and clinical integration. Image Vision Comput. (2025) 162:105708. doi: 10.1016/j.imavis.2025.10570842574925

[17] Aldakhil LA Alhasson HF Alharbi SS Khan RU Qamar AM . Image-Based Breast Cancer Histopathology Classification and Diagnosis Using Deep Learning Approaches. Appl Comput Intell Soft Comput. (2025) 2025, 7011984. doi: 10.1155/acis/7011984

[18] Pradeepa M Sharmila B Nirmala M . A hybrid deep learning model EfficientNet with GRU for breast cancer detection from histopathology images. Sci Rep. (2025) 15:24633. doi: 10.1038/s41598-025-00930-6 40634313 PMC12241594

[19] Arshad W Masood T Shahzad HM Ahmed HA Ahmed SH Khushi HMT . HistoDX: Revolutionizing Breast Cancer Diagnosis Through Advanced Imaging Techniques. IEEE Access. (2025) 13:94416–36. doi: 10.1109/ACCESS.2025.357421025079929

[20] Akbarnejad A Ray N Barnes PJ Bigras G . Toward accurate deep learning-based prediction of Ki67, ER, PR, and HER2 status from H&E-stained breast cancer images. Appl Immunohistochem Mol Morphol. (2025) 33:131–41. doi: 10.1097/pai.0000000000001258 40143808

[21] Shamai G Schley R Cretu A Neoran T Sabo E Binenbaum Y . Clinical utility of receptor status prediction in breast cancer and misdiagnosis identification using deep learning on hematoxylin and eosin-stained slides. Commun Med(2024) 4:276. doi: 10.1038/s43856-024-00695-5 39706861 PMC11661999

[22] Bai B Yang X Li Y Zhang Y Pillar N Ozcan A . Deep learning-enabled virtual histological staining of biological samples. Light Sci Appl. (2023) 12:57. doi: 10.1038/s41377-023-01104-7 36864032 PMC9981740

[23] Zhang Y Huang L Liu T Cheng K de Haan K Li Y . Virtual staining of defocused autofluorescence images using deep neural networks. Intell Comput. (2022) 2022:9818965. doi: 10.34133/2022/981896542641076

[24] Peng Q Lin W Hu Y Bao A Lian C Wei W . Advancing H&E-to-IHC virtual staining with task-specific domain knowledge for HER2 scoring. MICCAI 2024. (2024) 15004:3–13. doi: 10.1007/978-3-031-72083-3_128118817

[25] Ma J Li W Li J Liu Z Wu L Zhou F . Generative AI for misalignment-resistant virtual staining. Nat Commun. (2026) 17:71038. doi: 10.1038/s41467-026-71038-2 41888143 PMC13187172

[26] He K Zhang X Ren S Sun J . Deep residual learning for image recognition. Proc IEEE CVPR. (2016), 770–8. doi: 10.1109/cvpr.2016.9025079929

[27] Tan M Le QV . EfficientNet: Rethinking model scaling for CNNs. Proc ICML. (2019) 97, 6105–14.

[28] Lu MY Williamson DFK Chen TY Chen RJ Barbieri M Mahmood F . Data-efficient and weakly supervised computational pathology on WSIs. Nat BioMed Eng. (2021) 5:555–70. doi: 10.1038/s41551-020-00682-w 33649564 PMC8711640

[29] Paszke A Gross S Massa F Lerer A Bradbury J Chanan G . PyTorch: An imperative style, high-performance deep learning library. NeurIPS. (2019) 32:8024–35. doi: 10.5555/3454287.3455008

[30] Kingma DP Ba J . Adam: A method for stochastic optimization. ICLR. (2015).

[31] Goodfellow I Pouget-Abadie J Mirza M Xu B Warde-Farley D Ozair S . Generative adversarial nets. NeurIPS. (2014) 27:2672–81. doi: 10.1145/3422622

[32] Isola P Zhu JY Zhou T Efros AA . Image-to-image translation with conditional adversarial networks. Proc IEEE CVPR. (2017), 1125–34. doi: 10.1109/cvpr.2017.63225079929

[33] Zhu JY Park T Isola P Efros AA . Unpaired image-to-image translation using cycle-consistent adversarial networks. Proc IEEE ICCV. (2017), 2223–32. doi: 10.1109/iccv.2017.24425079929

[34] Rivenson Y Wang H Wei Z de Haan K Zhang Y Wu Y . Virtual histological staining of unlabelled tissue-autofluorescence images via deep learning. Nat BioMed Eng. (2019) 3:466–77. doi: 10.1038/s41551-019-0362-y 31142829

[35] Shafique A Babaie M Sajadi M Batten A Skdar S Tizhoosh HR . Automatic multi-stain registration of whole slide images in histopathology. In: IEEE EMBC (Piscataway, NJ: Institute of Electrical and Electronics Engineers (IEEE)), (2021). 10.1109/EMBC46164.2021.9629970 34892022

[36] Rombach R Blattmann A Lorenz D Esser P Ommer B . High-resolution image synthesis with latent diffusion models. In: Proc. IEEE/CVF CVPR (Los Alamitos, CA: IEEE Computer Society), (2022). p. 10684–95.

[37] Chen RJ Ding T Lu MY Williamson DFK Jaume G Song AH . Towards a general-purpose foundation model for computational pathology. Nat Med. (2024) 30:850–62. doi: 10.1038/s41591-024-02857-3 38504018 PMC11403354

[38] Lu MY Chen B Williamson DFK Chen RJ Liang I Ding T . A visual-language foundation model for computational pathology. Nat Med. (2024) 30:863–74. doi: 10.1038/s41591-024-02856-4 38504017 PMC11384335

